# Health in Factories

**Published:** 1920-01-31

**Authors:** 


### HEALTH IN FACTORIES.

### Steps by Enlightened Employers.

Between two and three hundred large firms have appointed a whole-time official to look after the welfare of their employees. This fact is related in an article by Sir Kingsley Wood, Parliamentary Secretary to the Minister of Health, published in the *Daily Express*. As he points out, health in the workshop is very largely regulated by the Factory Acts, but not entirely.

It is provided, for instance, that a boy just beginning work must undergo initial medical examination, but it may be something of a merely formal affair, and one of the duties of the whole-time officials, which these two or three hundred firms have appointed, is to attend this examination and to continue an interest in the boy's physical progress. These officials also organise all kinds of sport, encourage gardening, and manage field rambles or summer camps. It is an enlightened as well as a sensible economic step, for it pays to have healthy workmen. Shipbuilders, dyers, engineers, mineowners, manufacturers of all descriptions agree that better health means better time-keeping, but beyond all that, better health is an end in itself to be encouraged in every possible way. There is no arbitrary interference with men's lives or leisure in the scheme, which is purely voluntary and intended to give an organised lead to those sensible enough to follow. Sir Kingsley Wood's appeal to all employers to adopt it deserves backing.

In this connection it is well to know that there exists a newly-formed welfare workers' institute, with headquarters in Adelphi, representing about a thousand welfare workers attached to industrial undertakings, and engaged in the study and organisation of this kind of work. There should be much scope for it.

